# Inflamação pós-Infarto Agudo do Miocárdio: “Médico ou Monstro”

**DOI:** 10.36660/abc.20201250

**Published:** 2020-12-01

**Authors:** Ricardo Wang, Fernando Carvalho Neuenschwander, Bruno Ramos Nascimento

**Affiliations:** 1 Instituto Orizonti Departamento de Cardiologia Belo HorizonteMG Brasil Instituto Orizonti – Departamento de Cardiologia,Belo Horizonte, MG - Brasil; 2 Hospital das Clínicas Universidade Federal de Minas Gerais Belo HorizonteMG Brasil Hospital das Clínicas da Universidade Federal de Minas Gerais (UFMG), Belo Horizonte, MG - Brasil; 3 Hospital Unimed Belo Horizonte Belo HorizonteMG Brasil Hospital Unimed Belo Horizonte, Belo Horizonte, MG - Brasil; 4 Universidade Federal de Minas Gerais Belo HorizonteMG Brasil Universidade Federal de Minas Gerais, Belo Horizonte, MG – Brasil

**Keywords:** Infarto do Miocárdio, Inflamação/complicações, Linfócitos Tipo B, Citocinas, Vasoplegia, Choque Cardiogênico

Assim como no conto “Estranho Caso de Dr. Jekyll e Mr. Hyde”, a inflamação tem seu lado bom, que é a proteção contra patógenos, e ajuda no processo de reparação celular e tecidual após uma injúria; por outro lado, ela também pode perpetuar e amplificar a injúria e, no caso do infarto do agudo do miocárdio (IAM), pode ser o gatilho da oclusão coronariana. Na fase aguda do IAM, há ativação do sistema imunológico, no processo de reparação miocárdica, na qual o tecido necrosado é substituído pelo tecido cicatricial (fibrose). Sabemos, através dos estudos anatomopatológicos, que já nas primeiras horas pós-oclusão coronariana, há recrutamento principalmente de neutrófilos para o local da injúria. A população de neutrófilos local tem pico por volta do terceiro dia e em seguida observa-se um declínio progressivo. Eles são substituídos a partir do quinto dia por macrófagos, e ambos são responsáveis pelo processo de depuração de miócitos não viáveis. Os macrófagos, além deste papel, são responsáveis, juntamente com as células musculares lisas, pela angiogênese e produção de colágeno. O processo de cicatrização começa na periferia da área infartada e se estende para o núcleo, e este mecanismo de reparação se completa em torno de 4–8 semanas, dependendo do tamanho do infarto.^1 , 2^

Se sabemos como ocorre o processo inflamatório a nível celular no pós-IAM, qual o motivo de continuarmos estudando a inflamação? E por que concentrar os esforços nos estudos sobre a expressão de citocinas? Se, por um lado, o processo inflamatório é necessário para o processo de reparação, no contexto do IAM a inflamação também tem papel importante nas complicações. Tal efeito é observado no choque cardiogênico (provocando vasodilatação, vasoplegia e piora do choque),^3^ complicações mecânicas (ruptura de músculo papilar e da parede livre do ventrículo, e comunicação interventricular), no remodelamento ventricular (expansão e substituição fibrótica da parede acometida) e, a longo prazo, tem sido relacionado com a ocorrência de novos eventos cardiovasculares. As citocinas são moléculas que mediam reações imunes e inflamatórias e são responsáveis pela ativação de vias inapropriadas ou respostas exageradas (hipersensibilidade).^4^ Portanto, o entendimento de sua cinética pode ajudar a esclarecer as vias que estão associadas com desfechos favoráveis e as vias que, quando ativadas, podem levar a aumento de eventos desfavoráveis e têm potencial para serem alvos de futuras abordagens terapêuticas.

Na elegante subanálise do estudo BATTLE-AMI (Avaliação dos Linfócitos Tipos B e T no Infarto Agudo do Miocárdio), conduzido por Maria Coste e colaboradores,^5^ o objetivo principal foi estudar o comportamento do sistema imune durante a fase precoce e tardia do IAM tentando correlacionar com a área sob risco no IAM. Para isso, foram coletadas amostras de sangue de 138 pacientes (dentre os 300 participantes da amostra original do estudo), e foram dosadas citocinas pró-inflamatórias IL-1β (IL – Interleucina), IL-4, IL-6 e IL-18, e anti-inflamatória IL-10. Como era de se esperar, nos primeiros dias prevaleceram as citocinas pró-inflamatórias (IL-1β e IL18), e após quatro semanas observaram-se quedas das citocinas pró e aumento daquelas associadas a um perfil anti-inflamatório (IL-10). Mas os níveis de IL-4 e IL-6 se mantiveram elevados. Subanálises devem ser analisadas com cuidado e, devido ao risco do erro tipo I, elas têm primordialmente um papel gerador de hipóteses.^6^ Nesse estudo, menos da metade dos pacientes da amostra original foi analisada, e observamos uma média de idade mais baixa que a da literatura, e, desta forma, a resposta imunológica observada poderia ser diferente com a ampliação da amostra.^7^ Essa possibilidade é ainda mais possível se considerarmos a significativa variabilidade nos valores medidos das citocinas.

As análises múltiplas também devem ser olhadas com muito cuidado, principalmente quando se tem uma amostra limitada. No presente subestudo, foram correlacionados os níveis das citocinas com três variáveis da ressonância miocárdica, o que aumenta a possibilidade de o achado ter sido ao acaso. Isso poderia explicar, por exemplo, a correlação negativa entre a IL6 e a medida de fração de ejeção do ventrículo esquerdo, contudo sem associação com a massa do VE acometida no IAM.^5^ Como a área de necrose era de moderado tamanho (em torno de 10% no realce tardio), provavelmente poucos pacientes apresentaram choque cardiogênico e/ou remodelamento com expansão ventricular. Seria interessante avaliar, adicionalmente, o comportamento dessas citocinas nessas situações clínicas. Os dados, entretanto, acrescentam-se à literatura até então disponível, e a sua grande contribuição é tentar correlacionar as principais citocinas – já amplamente estudadas na fase aguda do IAM – com dados da ressonância cardíaca.

O estudo da inflamação tecidual tem chamado atenção principalmente após o estudo CANTOS ( *Canakinumab Antiinflammatory Thrombosis Outcome Study* ),^8^ no qual o bloqueio da IL-1β esteve associado com redução de eventos cardiovasculares ( *hazard ratio* 0,83; IC 95%, 0,73–0,95; p=0,005) em pacientes pós-IAM. Recentemente, o estudo COLCOT ( *Colchicine Cardiovascular Outcomes Trial* ),^9^ demonstrou que a cochicina (bloqueio inespecífico e difuso da inflamação) reduziu o desfecho primário composto (morte, IAM, parada cardíaca, acidente vascular encefálico, e hospitalização de urgência) ( *hazard ratio* 0,77; IC 95%, 0,61–0,96; p=0,02). Neste caminho, a IL-6 tem recebido especial interesse,^10 - 12^ pois seus níveis elevados estão associados com ativação de macrófagos, liberação de proteína C reativa, ativação de células musculares lisas e ação no metabolismo dos lípides – processos classicamente associados aos eventos coronarianos agudos. Dados indiretos, principalmente advindos de pacientes portadores de artrite reumatoide, sugerem que a elevação da IL-6 poderia ser o elo entre esta doença e eventos cardiovasculares.

Após demonstrada, a partir destes estudos, a relação da IL-6 com eventos cardiovasculares, o próximo passo é a realização de ensaios clínicos específicos. A tolicizumabe é um anticorpo monoclonal que bloqueia especificamente a IL-6,^13^ e tem se mostrada benéfica em pacientes portadores de artrite reumatoide, mas, por outro lado, foi ineficaz na fase aguda da infecção pelo vírus responsável pela SARS-COV-19 (durante a chamada “tempestade de citocinas”).^14^ Ainda não sabemos quais seriam as consequências de seu bloqueio no sistema cardiovascular. Apesar de estar muito associada com efeitos pró inflamatórios, a IL-6 também pode ter efeitos anti-inflamatórios.^4^ Como no conto de Stevenson, para acabar com o monstro, o médico Jerkill matou o hospedeiro. Temos que ter cuidado ao manipular o sistema imunológico, buscando efeitos benéficos como um melhor remodelamento ventricular e prevenção secundária de eventos pois, sem o conhecimento de todas as consequências e na ausência de evidências clínicas robustas, poderemos matar mais do que salvar.^15^
