# Supplementary material for: Inflamação pós-Infarto Agudo do Miocárdio: “Médico ou Monstro”
Source: Arq Bras Cardiol. 2020 Dec 1;115(6):1112–3. [Article in Portuguese] doi: 10.36660/abc.20201250 (PMC8133729; doi:10.36660/abc.20201250)
Supplement: Translation

# Translation: Inflammation Post-Acute Myocardial Infarction: “Doctor or Monster”

**Authors:** Ricardo Wang, Fernando Carvalho Neuenschwander, Bruno Ramos Nascimento

**Affiliations:** 1 Brazil Instituto Orizonti – Departamento de Cardiologia, Belo Horizonte, MG - Brazil; 2 Brazil Hospital das Clínicas da Universidade Federal de Minas Gerais (UFMG), Belo Horizonte, MG - Brazil; 3 Brazil Hospital Unimed Belo Horizonte, Belo Horizonte, MG - Brazil; 4 Brazil Universidade Federal de Minas Gerais, Belo Horizonte, MG – Brazil

**Keywords:** Myocardial Infarction, Inflammation/complications, B-Lymphocytes, Cytokines, Vasoplegia, Shock, Cardiogenic

As in the short story “The Strange Case of Dr. Jekyll and Mr. Hyde ”, inflammation has a good side, which is the protection against pathogens, and helps in the process of cellular and tissue repair after an injury; on the other hand, it may also perpetuate and worsen the injury and, in the case of acute myocardial infarction (AMI), it may be the trigger of coronary occlusion. In the acute phase of AMI, the immune system is activated in the process of myocardial repair, in which the necrotic tissue is replaced by the scar tissue (fibrosis). From anatomopathological studies, we know that in the first hours after coronary occlusion, neutrophils are mainly recruited to the injury site. The local neutrophil population peaks around the third day, then a progressive decline is observed. From the fifth day, they are replaced by macrophages and both are responsible for clearing non-viable myocytes. In addition to this role, together with smooth muscle cells macrophages are responsible for angiogenesis and collagen production. The scarring process begins at the periphery of the infarcted area and extends to the nucleus, and this repair mechanism is completed in about 4–8 weeks, depending on the infarction size.^1 , 2^

If we know how the inflammatory process occurs at the cellular level post-AMI, why do we continue to study inflammation? And why concentrating efforts on studies on cytokine expression? Whereas, on the one hand, the inflammatory process is necessary for the repair process, in the context of AMI, inflammation also plays an important role in complications. Such effect is observed in cardiogenic shock (causing vasodilation, vasoplegia and worsening shock),^3^mechanical complications (papillary muscle rupture and ventricular free wall, and interventricular communication), in ventricular remodeling (fibrotic expansion and replacement of the affected wall) and, in the long run, it has been related to new cardiovascular events. Cytokines are molecules that mediate immune and inflammatory reactions and are responsible for activating inappropriate pathways or exaggerated responses (hypersensitivity).^4^Therefore, understanding its kinetics can help to clarify the pathways associated with favorable outcomes and the pathways that, when activated, may lead to an increase in unfavorable events and have the potential to be the target of future therapeutic approaches.

In the elegant subanalysis of the BATTLE-AMI study (B and T Types of Lymphocytes Evaluation in Acute Myocardial Infarction), conducted by Maria Coste et al.^5^the main objective was to study the behavior of the immune system during the early and late phase of AMI trying to correlate it with the area at risk in AMI. For this, blood samples were collected from 138 patients (from among the 300 participants from the original study sample), and pro-inflammatory cytokines IL-1β (IL – Interleukin), IL-4, IL-6 and IL-18, and anti-inflammatory IL-10 were dosed. As expected, pro-inflammatory cytokines (IL-1β and IL18) prevailed in the first days and, after four weeks, pro-cytokine declines were observed and an increase in those associated with an anti-inflammatory profile (IL-10). But the levels of IL-4 and IL-6 remained high. Subanalyses should be analyzed carefully and, due to the risk of type I error, they have a primary role in generating hypotheses.^6^In this study, less than half of the patients in the original sample were analyzed, and we observed a lower average age than that found in the literature, and, thus, the immune response observed could be different with the expansion of the sample.^7^This possibility is even more possible if we consider the significant variability in the measured values of cytokines.

Multiple analyses should also be looked at very carefully, especially when you have a limited sample. In the present sub-study, cytokine levels were correlated with three myocardial resonance variables, which increases the possibility that the finding had been by chance. This could explain, for example, the negative correlation between IL6 and the left ventricular ejection fraction, but without an association with the LV mass affected in AMI.^5^As the area of necrosis was of moderate size (around 10% in late enhancement), probably few patients had cardiogenic shock and/or remodeling with ventricular expansion. It would be interesting to further evaluate the behavior of cytokines in these clinical situations. The data, however, add to the literature hitherto available, and their major contribution is to try to correlate the main cytokines — already widely studied in the acute phase of AMI — with cardiac resonance data.

The study of tissue inflammation has drawn attention mainly after the CANTOS study (Canakinumab Antiinflammatory Thrombosis Outcome Study),^8^in which IL-1 block was associated with a reduction in cardiovascular events (hazard ratio 0.83; 95% CI, 0.73–0.95; p=0.005) in post-AMI patients.β Recently, the COLCOT study (Colchicine Cardiovascular Outcomes Trial)^9^demonstrated that colchicine (non-specific and diffuse inflammation block) reduced the primary composite outcome (death, AMI, cardiac arrest, stroke and emergency hospitalization) (hazard ratio 0.77; 95% CI, 0.61–0.96; p=0.02). IL-6 has thus received special interest,^10 - 12^because its high levels are associated with activation of macrophages, release of C-reactive protein, activation of smooth muscle cells and action on lipid metabolism — processes classically associated with acute coronary events. Indirect data, mainly from patients with rheumatoid arthritis, suggest that the increase of IL-6 could be the link between this disease and cardiovascular events.

As these studies demonstrated the relationship of IL-6 with cardiovascular events, the next step is to carry out specific clinical trials. Tolicizumab is a monoclonal antibody that specifically blocks IL-6,^13^and has been shown to be beneficial for patients with rheumatoid arthritis, but, on the other hand, it was ineffective in the acute phase of infection by the SARS-COV-19 virus (during the so-called “cytokine storm”).^14^We do not yet know what the consequences of its blockage on the cardiovascular system would be. Although it is highly associated with pro-inflammatory effects, IL-6 can also have anti-inflammatory effects.^4^As in Stevenson’s tale, to get rid of the monster, doctor Jerkill killed the host. We have to be careful with the immune system, looking for beneficial effects such as better ventricular remodeling and secondary prevention of events because, without knowing all the consequences and in the absence of robust clinical evidence, we might kill more than save.^15^
